# Catalytic conversion of lignin pyrolysis model compound- guaiacol and its kinetic model including coke formation

**DOI:** 10.1038/srep37513

**Published:** 2016-11-21

**Authors:** Huiyan Zhang, Yun Wang, Shanshan Shao, Rui Xiao

**Affiliations:** 1 Ministry of Education of Key Laboratory of Energy Thermal Conversion and Control, School of Energy and Environment, Southeast University, Nanjing 210096, P.R. China

## Abstract

Lignin is the most difficult to be converted and most easy coking component in biomass catalytic pyrolysis to high-value liquid fuels and chemicals. Catalytic conversion of guaiacol as a lignin model compound was conducted in a fixed-bed reactor over ZSM-5 to investigate its conversion and coking behaviors. The effects of temperature, weight hourly space velocity (WHSV) and partial pressure on product distribution were studied. The results show the maximum aromatic carbon yield of 28.55% was obtained at temperature of 650 °C, WHSV of 8 h^−1^ and partial pressure of 2.38 kPa, while the coke carbon yield was 19.55%. The reaction pathway was speculated to be removing methoxy group to form phenols with further aromatization to form aromatics. The amount of coke increased with increasing reaction time. The surface area and acidity of catalysts declined as coke formed on the acid sites and blocked the pore channels, which led to the decrease of aromatic yields. Finally, a kinetic model of guaiacol catalytic conversion considering coke deposition was built based on the above reaction pathway to properly predict product distribution. The experimental and model predicting data agreed well. The correlation coefficient of all equations were all higher than 0.90.

With the increasing concerns about energy shortage and environmental threats caused by large-scale consumption of fossil fuels, the utilization of biomass resources, which are the only renewable resource of carbon and available for the production of liquid carbon-based fuels and chemicals, is expected to grow fast in the coming years^1^,^2^,^3^,^4^. Catalytic pyrolysis is regarded as one of the most effective technologies to convert solid biomass to high-value liquid fuels and chemicals directly in one step^5^,^6^,^7^,^8^,^9^,^10^.

Lignin is one of the three main components of biomass (15–20%), including two types of aromatic nuclei, guaiacyl (4-hydroxy-3-methoxyphenyl) and syringyl (3,5-dimethoxy-4-hydroxyphenyl) units^11^,^12^. It has attracted considerable interests as a potential source of aromatic hydrocarbons for biofuels and chemicals due to its aromatic nature^13^,^14^. The conversion of lignin into useful chemical compounds has being a challenge for a long time not only because its resistant structure but also for the serious coke deposition^15^,^16^. The presence of oxygenates, such as guaiacol or phenol, contributes to the formation of coke^17^. Gayubo *et al.*^18^,^19^,^20^ found that phenols were the main precursors of coke and contribute to the catalyst deactivation. It is of great significance to suppress the coke formation and improve the yield of aromatics.

Some researchers have used modified catalysts like metal doped catalysts, pore modified catalysts aiming at changing catalysts acid property and pore structure to increase hydrocarbon yield^21^. Zheng *et al.*^22^ showed that ZSM-5 with crystal size of 200 nm exhibited the maximum aromatic yield and minimum BTX selectivity. Vichaphund *et al.*^23^ found that the addition of synthesized metal/HZSM-5 improved the aromatic selectivity up to 91–97% and decreased the undesirable oxygenated (0.6–4.0%) and N-containing compounds (1.8–4.6%). However, the problems of coke deposition which resulted in catalyst rapid deactivation and the reduction of the yields of targeted olefins and aromatics are still exited. Thus, the investigations for the reaction chemistry and mechanism of coking deactivation are necessary to increase the yields of hydrocarbons and find an efficient method to prevent the coke deposition.

Some studies about lignin catalytic pyrolysis have been performed^24^,^25^. However, the literatures involving detail mechanism features and coking characteristics are scarce. The catalytic pyrolysis of lignin is an extremely complex chemical process, in which fundamental basis is typically studied using simplified models^26^,^27^,^28^. It is acknowledged that coke deposition has a great influence on product distribution^29^,^30^,^31^. As coke content is easily to obtain online, it is significant to build a kinetic model considering coke deposition which could help to understand the quantitative relationship between coke content and product distribution. Many papers have reported about the kinetic model of biomass pyrolysis in recent years^32^. A method called “species lumps” which lumps the products of same species together with simplifying the product species is widely used in methanol-to-olefins (MTO)^33^,^34^,^35^. However, the coking rate for lignin catalytic pyrolysis is ten times more than that in MTO, the kinetic model for MTO is not suitable for the process. Thus it has great significance to build a kinetic model considering coke deposition for lignin catalytic conversion.

In this work, guaiacol is chosen as lignin model compound in order to investigate the complicated chemistry of lignin catalytic conversion. Catalytic conversion of guaiacol was conducted systematically in a fixed-bed reactor over ZSM-5 to investigate its conversion and coking behaviors. The effect of temperature, weight hourly space velocity (WHSV) and partial pressure on product distribution was studied. A possible reaction pathway was proposed based on the experimental data. Furthermore, the behaviors of coke deposition were investigated through a series of characterization methods. A lumped kinetic model including the deactivation caused by coke deposition was developed to predict the product distribution.

## Results

### The product distribution of guaiacol catalytic conversion

Different temperature, WHSV, partial pressure and time on stream (TOS) were tested to investigate their effects on product distribution. Table 1 listed the carbon yields of products obtained from guaiacol catalytic pyrolysis at all reaction conditions. The products were grouped into five different groups: One carbon products (CH_4_/CO/CO_2_), olefins (C_2_–C_4_), aromatics, phenols and coke. The carbon yields of olefins, aromatics, one carbon products and coke increased obviously as the temperature increasing from 500 to 650 °C, whereas the carbon yield of phenols initially increased and then declined. It indicated that low temperature favored to the pyrolysis of guaiacol to phenols, while high temperature promoted the production of olefins and aromatics, but also resulted in higher coke formation. With the increase of WHSV from 4 to 10.7 h^−1^, the carbon yield of coke decreased significantly, which was caused by the reduction of reactants retention time in the catalyst bed. While the reactant contact time with catalyst bed was raised by the increase of partial pressure, so the carbon yields of coke increased dramatically. The maximum aromatic carbon yield of 28.55% was obtained under the optimizing conditions: temperature, 650 °C; WHSV, 8 h^−1^; partial pressure, 2.38 kPa. The carbon yields of one carbon products, olefins and aromatics decreased significantly with increasing TOS, whereas phenols increased, which indicated that quick catalyst deactivation caused by coke deposition took place.

### The coke characteristics in catalytic pyrolysis of guaiacol

**Figure 1:**
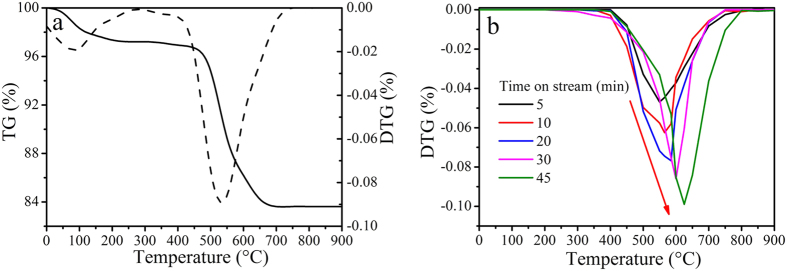
Thermogravimetric analysis of coked catalysts (**a**) time on stream: 30 min; (**b**) DTG for different reaction times.

Thermogravimetric Analysis (TGA) was used to measure the amount of coke deposited on the catalyst under different reaction time, and the results were shown in Fig. 1. There are two distinct weight loss steps during the process of thermal analysis of the coked catalyst running for 30 minutes in Fig. 1(a). The first weight loss occurred during room temperature to 300 °C could correspond to the desorption of moisture and physical adsorbents; the second one in the 400–800 °C range was attributed to the combustion of coke deposited on the catalyst which was usually defined as coke content of the catalyst. Figure 1(b) showed the DTG curves of coked catalysts with different reaction time. It was observed that the coke combustion peak moved to higher temperature as increasing reaction time, which indicates that harder combustion components were formed at long reaction time.

The surface area and pore volume of the fresh and deactivated catalysts were measured to analyze the effects of coke on catalyst texture using Nitrogen Isothermal Adsorption Desorption Analysis. The results were summarized in Table 2. The BET surface area dropped almost 75% after being used for 45 min, and the pore volume underwent similar decay, decreasing from 0.338 to approximately 0.141 cm^3^·g^−1^. It revealed that coke deposited on the catalyst surface and blocked the pores during the catalytic pyrolysis of guaiacol.

**Figure 2:**
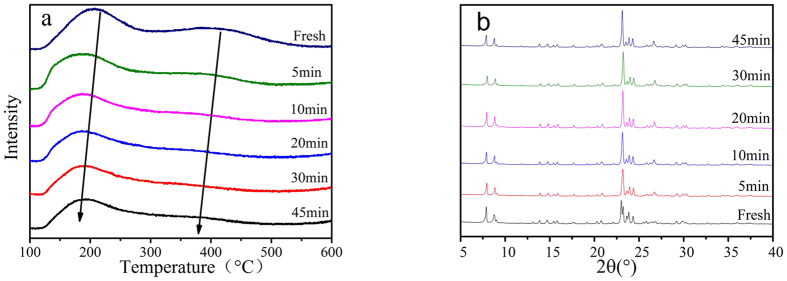
(**a**) NH_3_-TPD curves of the fresh and coked catalysts; (**b**) XRD patterns of the fresh and coked catalysts under different reaction times.

The NH_3_-Temperature-Programmed Desorption (NH_3_-TPD) Analysis was used to investigate the effects of coke deposition on the quantity and strength distributions of ZSM-5 acid sites at different reaction times. Figure 2(a) presented the NH_3_-TPD curves of fresh and coked catalysts. It can be seen from the figure, two desorption peaks assigned to weak (100–300 °C) and strong acid sites (300–500 °C) were recognized, respectively. The area of peak could represent the number of acid sites. It could be found that the strong acid sites decreased sharply, while the weak acid sites decreased slightly. When the catalyst was used for 45 min, most of the strong acid sites disappeared. The notable reduction of acid sites was attributed to coke deposition, and the coke generated preferentially on the strong acid sites. It can also be noted that the positions of desorption peaks shifted to lower temperature with increasing reaction time, which indicates the strength of acid sites decreased.

The X-ray Diffraction (XRD) patterns of the fresh and coked catalysts in the angle region of 2θ = 5–40° were measured to identify the coke deposition location. As shown in Fig. 2(b), both fresh and coked catalysts were detected as the crystalline phase with characteristic peaks at 2θ = 7.8, 8.7, 22.9, 23.8 and 24.2°, indicating the catalysts were in a well crystallized state. However, the intensity of peak at 2θ = 22.9° increased and the relative position moved slightly to higher angle. The characteristic diffraction reflection at 2θ = 26.8° was belonged to carbon appeared in the patterns of coked catalysts. The changes in the relative positions and intensities of the peaks in the XRD spectra of the coked catalyst could be related to the lattice deformation of the ZSM-5 caused by the deposition of carbonaceous materials inside zeolite pores^36^. It could be speculated that there were some coke deposited inside the channels of the zeolite after being run for 10 min, and its amount increased with the prolonging of the TOS from 10 to 45 min.

**Figure 3:**
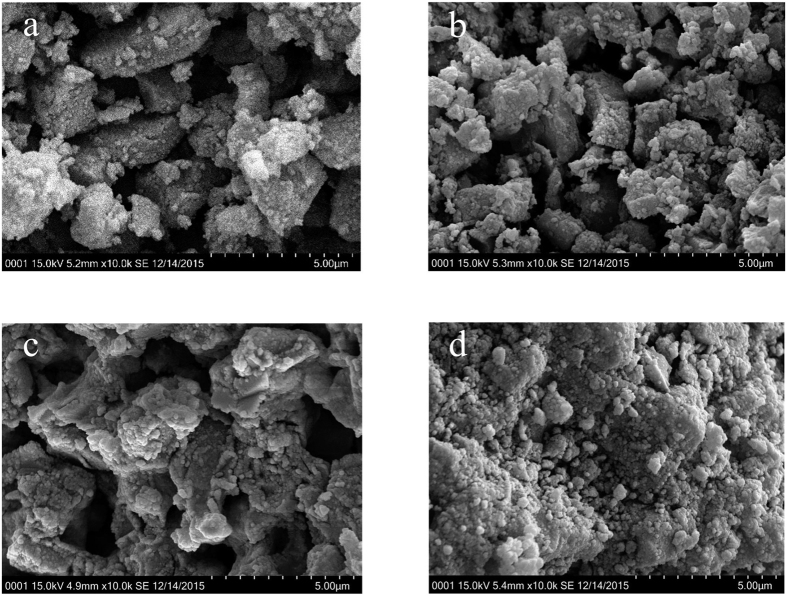
SEM images of the fresh catalyst (**a**) and coked catalysts at 5 min (**b**) 20 min (**c**) 45 min (**d**).

Scanning Electron Microscope (SEM) images of fresh and used catalysts (5, 20, 45 min) were tested to examine the morphology of coke on the deactivated catalysts, and the results were shown in Fig. 3. It could be found that some spherical particles which identified as carbon-rich large molecules generated and accumulated rapidly on the catalyst surfaces. With increasing reaction time, the active sites were covered and the catalyst pore were also blocked which caused the deactivation of catalyst.

## Discussion

### The conversion pathway of guaiacol to produce aromatics and olefins

**Figure 4:**
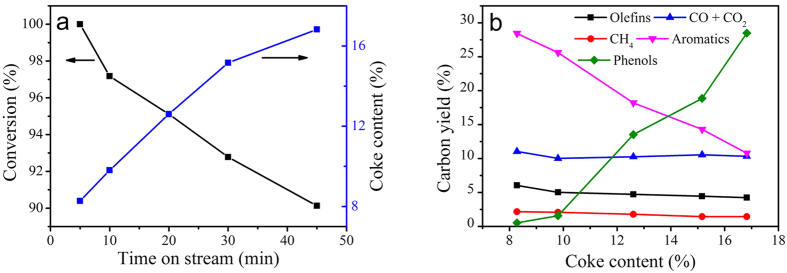
(**a**) Conversion and coke content Venus time on stream during catalytic conversion of guaiacol over ZSM-5; (**b**) effect of coke content on the product carbon yield.

Figure 4(a) showed the evolution of conversion and coke content with time on stream of conversion under the following reaction conditions: temperature of 600 °C, guaiacol partial pressure of 2.38 kPa and WHSV of 8 h^−1^. It could be found that the conversion of guaiacol declined sharply from 100% to 90.1%, meanwhile the coke content increased. Table 3 displayed the carbon yields of specific quantified products versus TOS. The main composition of products were benzene, CO, ethylene, phenol, 2-Hydroxybenzaldehyde and 1,2-Benzenediol. At the last period of reaction time (30–45 min), phenols accounted for a large proportion of products.

The coke formation led to catalyst active sites coverage along with catalyst pores blockage, and thus the product yields were affected^37^,^38^. Figure 4(b) showed the relationship between carbon yields of products for guaiacol catalytic pyrolysis and the determined coke content. The aromatic carbon yield decreased from 28.42% at coke content of 8.2% rapidly to 10.8% at coke content of 16.8%, while that of phenols increased dramatically. The results revealed that coke deposition was responsible to the deactivation of catalyst.

**Figure 5:**
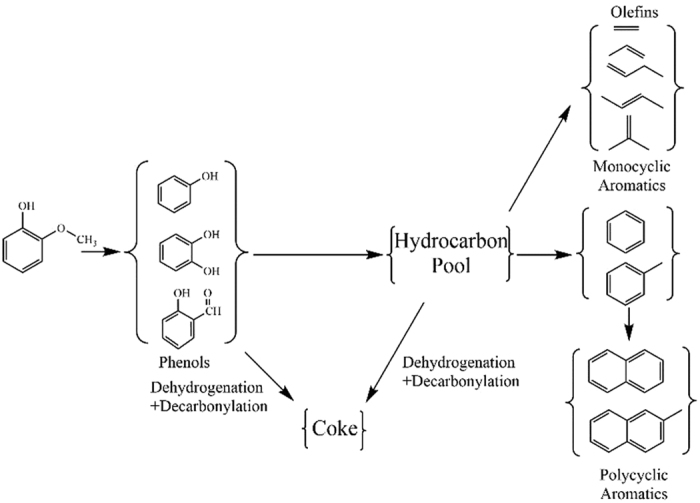
The catalytic conversion pathway of guaiacol.

The catalytic pyrolysis pathway of guaiacol was speculated as shown in Fig. 5. The methoxyl of guaiacol was unstable and easy to crack to form phenols^17^,^39^. These intermediate oxygenates further transformed into olefins and aromatics through bond breaking and aromatization. The coke might be formed through a series of decarboxylation and dehydrogenation during the reaction.

### Kinetic modeling of guaiacol catalytic conversion considering the deactivation by coke

In this work, it is expected to understand the relationship between product yield and coke content. The fixed-bed reactor is regarded as an ideal isothermal plug flow reactor and the coke content is regarded as distributed in the catalyst bed uniformly. The kinetic models have been proposed based on the following general expression for the reaction rate of each *i* component:


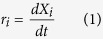


where *X*_*i*_ is the molar fraction of each lump on a CH_2_ basis.

The reaction network was established based on the pathway discussed above. Guaiacol was first converted to intermediate oxygenates with reaction rate of *k*_*5*_ and then converted to one carbon products (CO/CO_2_/CH_4_), olefins, aromatics and coke with reaction rate of *k*_*1*_, *k*_*2*_, *k*_*3*_ and *k*_*4*_, respectively. The feed and products have been divided into six lumps. Phenols are regarded as the important immediate product. All the hydrocarbons are formed mainly parallel as secondary products.

The expressions determined for the formation of each lump are as follows:


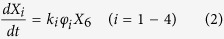



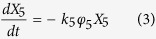



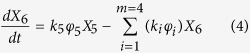


where i = 1, 2, 3, 4 and 5 represent for one carbon products, olefins, aromatics, coke and oxygenated intermediates, respectively.

Considering the quality of WHSV and partial pressure effect on the reaction rate, a modified Arrhenius equation has been proposed.


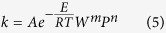


where *k* is the rate constant, *A* is the frequent factor, *E* is activation energy, *W* is WHSV, *P* is guaiacol partial pressure, *m* and *n* are the impact factors of WHSV and partial pressure.

All rate constants are supposed to rely on the coke content of catalyst, which is taken into consideration by the deactivation function 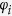. Different deactivation functions have been tested to describe the coke influence on reaction rates^31^. The best fit to the experimental data has been obtained with exponential function.


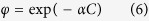


where C is the weight percent of coke on the catalyst (g_coke_/g_cat_%), α is inactivation constant. The different reaction steps have different α values. The effect of coke on the reaction rates is modeled by using different values for the empirical α constants.

A fourth-order Rung-Kutta method was used to integrate the ordinary differential equations. The kinetic parameters have been determined by multivariable non-linear regression following two successive routines: firstly, we use genetic algorithm to obtain a fast overall approximation to the optimum values of the parameters; secondly, taking these values as initial ones, nonlinear least-squares routine in MATLAB using the Levenberg-Marquardt method is used to obtain a close approximation to the optimum.

The kinetic parameters for each kinetic model proposed have been calculated by minimizing an objective function EOF, which is defined as the sum of squares of the differences between the experimental and calculated values of composition.


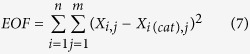


where index *i* presents the component; index *j*, the kinetic runs; 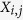 and 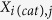 are experimental and calculated molar fraction of component *i*.

**Figure 6:**
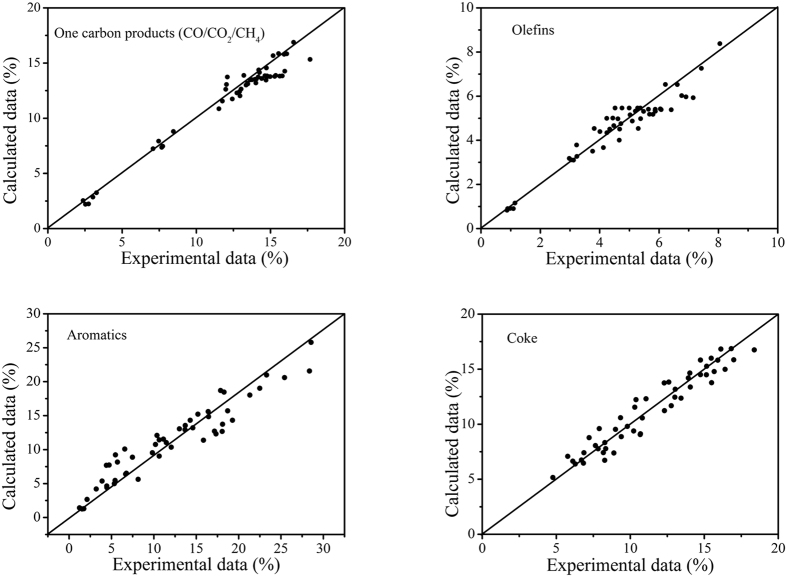
Comparison between experimental and calculated values.

The kinetic parameters including frequent factors, activation energies, inactivation constants and impact factors are listed in Table 4. The correlation coefficient of all equations were all higher than 0.90. The accuracy of the regression is tested by means of F-test. Statistical analysis indicated that the model is high creditable. Figure 6 compares the experimental results with those calculated ones using the kinetic model. The results showed the model fitted the experimental results well.

## Methods

### Materials

Guaiacol (99.0%, Aladdin Company, P.R. China) was used as the feedstock in this study. ZSM-5 catalyst with a SiO_2_/Al_2_O_3_ molar ratio of 38 was supplied by the catalyst plant of Nankai University. The catalyst was sieved to 80–100 mesh and calcined in oxygen flow at 600 °C for 6 h before all reactions.

### Catalytic conversion experiments

**Figure 7:**
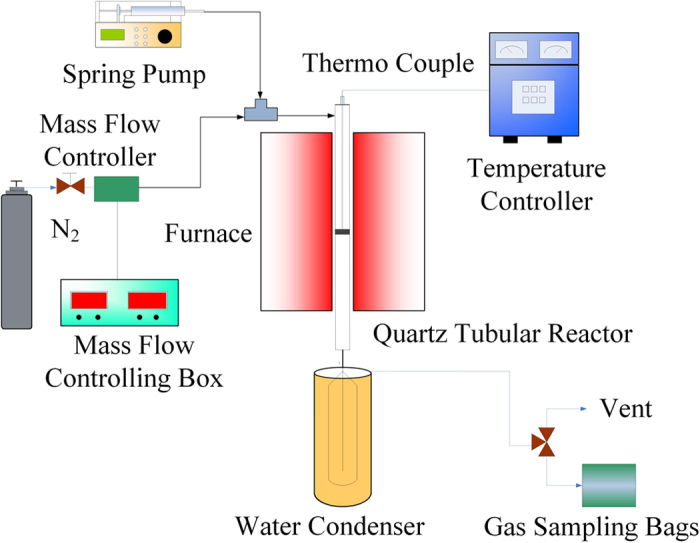
The schematic diagram of the fixed bed for catalytic conversion of biomass derivate.

The catalytic pyrolysis of guaiacol was carried out in a fixed-bed quartz reactor shown in Fig. 7. Sieved catalysts were held on the quartz bed. Guaiacol was pumped into the reactor by a spring pump at the top of the reactor, then vaporized and flew across the catalyst bed by the carrier gas (N_2_). The guaiacol catalytic pyrolysis was performed at temperature between 400 and 650 °C, WHSV ranging from 4 to 10.7 h^−1^, guaiacol partial pressure ranging from 0.98 to 3.12 kPa. The runs with different WHSV were carried out at 600 °C and a guaiacol partial pressure of 2.38 kPa, and the runs with different guaiacol partial pressure were also performed at 600 °C. Each run with different reaction condition was carried out for 5, 10, 20, 30 and 45 min. The condensed products were extracted by 10 mL of ethanol from the condensers to obtain the liquid products and analyzed by GC/MS (Agilent, 7890A-5975C). The gas products were collected in the gas sampling bags and analyzed by GC-FID/TCD (Shimadzu 2014). After reaction, the reactor was flushed with nitrogen of 100 mL/min for 30 min at the reaction temperature. When the reactor cooled to room temperature, the coked catalysts were taken out for different analyses.

### Characterizations

Coke amount deposited on the catalyst was measured by combustion of the deactivated catalyst taken from the fixed bed reactor using a thermogravimetric (TG) analyzer. The coked catalysts were heated from room temperature to 900 °C at a heating rate of 20 °C/min in an air stream.

The surface areas and pore volumes of the catalyst were measured by the nitrogen adsorption isotherms on an automatic volumetric adsorption measurement system (Gold APP Instruments, V-Sorb 2800 P, China). Samples were evacuated at 623 K for 3 h before exposing them to nitrogen gas at 77 K.

Ammonia temperature programmed desorption (NH_3_-TPD) was applied to study the surface acidity of the catalysts. 50 mg of the sample was pretreated in He stream (20 mL/min) at 550 °C for 60 min, cooled down to 100 °C and saturated with 5% ammonia-He to the equilibrium state; then, the sample was flushed with a He stream to remove physically adsorbed ammonia; finally, the NH_3_-TPD measurements were carried out from 100 to 600 °C with a heating rate of 10 °C/min.

X-ray diffraction (XRD) analysis was recorded on a Rigaku UItima IV diffractometer (Japan) with Cu KR radiation at 40 kV and 30 mA condition. Scans were taken at 5–40° 2θ range with a scanning rate of 5°/min.

Scanning electron microscope (Hitachi, SU3500, Japan) were performed to obtain the surface morphology of the coked catalysts.

### Statistical analysis

The calculations used in this paper are summarized in the following. The weight hour space velocity (WHSV) is defined as:


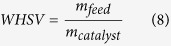


where 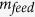 is mass flow rate of feed (mg·h^−1^), 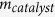 is mass of catalysts loaded on the plate (mg). The partial pressure of guaiacol is defined as:


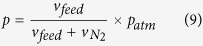


where 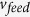 is flow rate of feed (mL·min^−1^), 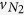 is flow rate of nitrogen (mL·min^−1^). 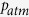 is the atmosphere pressure. The carbon yield of identified product is defined as:


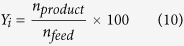


where 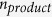 is moles of carbon in identified products (mol), 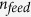 is moles of carbon in feed (mol). The amount of coke on the catalyst is given as:


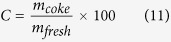


where *m*_*coke*_ is mass loss of catalyst in TG (mg), *m*_*fresh*_ is mass of fresh catalyst (mg).

## Additional Information

**How to cite this article**: Zhang, H. *et al.* Catalytic conversion of lignin pyrolysis model compound-guaiacol and its kinetic model including coke formation. *Sci. Rep.* **6**, 37513; doi: 10.1038/srep37513 (2016).

**Publisher’s note:** Springer Nature remains neutral with regard to jurisdictional claims in published maps and institutional affiliations.

## Figures and Tables

**Table 1:** The product distribution of guaiacol catalytic conversion at different reaction conditions.

T (°C)	WHSV (h^−1^)	P (kPa)	TOS (min)	Carbon yield (wt%)					
				One carbon products	Olefins	Aromatics	Coke	Intermediate Oxygenates	Guaiacol
500	8	2.38	5	3.28	1.14	4.46	10.49	0.20	5.03
			10	3.03	1.09	3.21	6.91	0.62	8.25
			20	2.74	0.99	2.12	4.30	2.77	23.23
			30	2.54	0.88	1.74	3.44	4.19	25.65
			45	2.38	0.90	1.24	3.01	10.68	26.63
550	8	2.38	5	8.46	3.23	13.70	15.03	0.65	4.49
			10	7.74	3.23	10.64	9.16	2.19	7.31
			20	7.67	3.12	6.67	5.94	6.63	12.27
			30	7.46	3.04	5.35	4.77	11.68	14.12
			45	7.09	2.98	4.45	3.60	21.07	16.71
600	4	2.38	5	16.10	8.05	18.28	25.38	3.63	6.19
			10	15.92	7.43	15.21	15.88	7.91	11.71
			20	15.18	6.61	10.64	11.33	18.83	16.16
			30	14.73	6.22	8.16	9.55	28.33	17.83
			45	14.21	5.87	5.47	7.39	35.77	18.66
600	6.4	2.38	5	14.77	5.88	22.45	18.87	2.09	3.49
			10	14.26	5.68	19.28	12.36	5.01	8.41
			20	13.63	4.61	17.33	7.60	10.99	13.09
			30	13.35	4.34	15.85	6.42	22.91	15.08
			45	12.92	3.76	12.06	4.72	29.21	16.71
600	8	0.98	5	16.56	5.37	14.29	13.44	0.64	4.67
			10	15.54	5.28	10.20	8.41	1.70	8.31
			20	14.63	4.99	5.71	5.62	2.09	15.27
			30	14.17	4.75	3.90	4.92	6.85	18.12
			45	13.76	4.52	1.53	3.83	14.80	23.71
600	8	1.92	5	15.97	7.13	21.32	14.74	0.61	4.49
			10	15.63	6.41	16.45	9.10	1.61	8.71
			20	14.71	5.80	11.48	5.71	7.40	13.84
			30	14.02	5.48	7.27	5.10	12.91	16.58
			45	13.03	5.20	4.41	3.89	18.78	21.71
600	8	2.38	5	13.21	6.05	28.42	18.19	0.53	2.83
			10	12.10	5.02	25.42	10.77	3.77	4.89
			20	12.04	4.72	18.07	6.93	14.90	7.22
			30	11.99	4.45	13.67	5.55	19.96	9.87
			45	11.76	4.24	10.37	4.11	28.48	12.94
600	8	3.15	5	15.79	5.38	18.71	17.98	1.22	5.14
			10	14.98	5.10	16.41	11.75	2.27	10.46
			20	13.95	4.67	6.57	7.02	4.63	15.26
			30	13.47	4.48	5.43	5.68	8.71	17.32
			45	12.95	4.24	4.73	4.15	15.78	18.26
600	10.67	2.38	5	14.41	5.30	23.31	13.03	3.80	2.73
			10	13.45	4.66	18.11	7.70	6.80	4.65
			20	12.74	4.12	17.16	5.07	12.48	7.06
			30	12.43	4.01	14.60	4.05	14.80	9.94
			45	11.53	3.82	13.03	2.95	15.87	12.19
650	8	2.38	5	17.67	6.91	28.55	19.55	0.63	0.00
			10	15.36	6.76	17.88	11.73	1.16	1.03
			20	15.29	6.07	11.14	7.72	5.72	4.73
			30	14.74	5.87	9.83	6.01	8.27	6.49
			45	14.05	5.64	6.77	4.49	12.82	8.89

**Table 2:** N_2_-physisorption of fresh and coked catalysts.

Time on stream (min)	Surface area (m^2^/g)	Pore volume (cm^3^/g)
0	302.588	0.338
5	215.819	0.249
10	191.887	0.23
20	141.388	0.198
30	120.847	0.183
45	75.605	0.141

**Table 3:** Carbon yields of products as a function of time on stream.

	Time on stream (min)				
	5	10	20	30	45
Ethylene	4.77	4.04	3.71	3.43	2.80
Propylene	1.14	0.78	0.71	0.64	0.40
Butene	0.14	0.21	0.30	0.37	1.04
CH_4_	2.17	2.09	1.78	1.44	1.43
CO	10.36	9.55	10.01	10.31	10.25
CO_2_	0.68	0.46	0.25	0.24	0.08
Benzene	17.74	15.40	11.56	8.40	6.44
Toluene	4.68	4.25	3.46	2.97	1.97
Naphthalene	6.00	5.77	3.05	2.31	1.96
Phenol	0.53	0.76	1.81	2.35	6.21
2-Hydroxybenzaldehyde	—	3.00	3.71	4.48	5.21
2-Methylphenol	—	—	0.40	0.57	0.64
2-Ethylphenol	—	—	—	—	0.19
1,2-Benzenediol	0.00	0.00	8.97	12.55	16.24
Guaiacol	2.82	4.89	7.22	9.87	12.94
Olefins	6.05	5.02	4.72	4.45	4.24
C1	13.21	12.09	12.04	11.99	11.76
Aromatics	28.42	25.42	18.07	13.67	10.37
Phenols	0.53	3.77	14.90	19.96	28.48
Coke	18.19	10.77	6.93	5.55	4.11

**Table 4:** Parameters for the kinetic model of guaiacol catalytic conversion.

k_i_	A	E (kJ/mol)	α	m	n
k_1_	7.68	94.37	43.69	2.68	2.91
k_2_	57.19	79.19	44.52	3.35	5.49
k_3_	158.37	71.94	30.58	0.25	2.05
k_4_	2712509.57	98.73	47.18	1.96	0.38
k_5_	114518.44	115.36	12.91	0.05	0.17
